# Dataset of quantification and classification of microplastics in Mexican sandy beaches

**DOI:** 10.1016/j.dib.2020.106473

**Published:** 2020-10-31

**Authors:** Juan Carlos Alvarez-Zeferino, Sara Ojeda-Benítez, Arely Areanely Cruz-Salas, Carolina Martínez-Salvador, Alethia Vázquez Morillas

**Affiliations:** a Universidad Autónoma de Baja California – Instituto de Ingeniería, UABC, Boulevard Benito Juárez y Calle de la Normal S/N, Col. Insurgentes Este, C.P. 21280 Mexicali, Baja California, México; b Universidad Autónoma Metropolitana - Azcapotzalco, Av. San Pablo, No. 180 Col. Reynosa-Tamaulipas, C.P. 02200 Ciudad de México, México; c MSc on Water Sciences and Engineering, IHE-UNESCO, Westvest 7, 2611 AX Delft, The Netherlands

**Keywords:** Classification of microplastics, Fragments, Pellets, colour, Extraction

## Abstract

The presence of microplastics in marine and coastal environments is an issue of concern for the preservation of these ecosystems. This dataset provides the results of the first national assessment of the presence of microplastics (<5 mm) in Mexican beaches. The research included 35 beaches along the five marine regions of the country. For each beach, ten samples (19 cm diameter, 5 cm depth) were taken along the high-tide line, and then microplastics were extracted by flotation and sieving. All the plastic particles were quantified and classified based on their colour, type of particle, and type of plastic used in their production, using visual inspection and FTIR spectroscopy. The use of the same methodology in all the beaches allowed us to compare and analyze the results, which are reported in the article Microplastics in Mexican beaches.

## Specifications Table

**Table:** 

Subject	Environmental Science (general)
Specific subject area	Coastal Management and Research
Type of data	Table
How data were acquired	Samples were taken directly in the 35 beaches included in the study. Identification of colour and type of microplastics: direct visual inspection Identification of type of plastics: Fourier transform infrared spectroscopy (FTIR), Spectrum Two FT-IRL-160000A Perkin Elmer
Data format	Raw
Parameters for data collection	Sandy beaches, with a minimal length of 100 m, and at least 1 km away from docks were selected for the study. Samples were taken during hurricane season (April-July 2018) in all the beaches. Ten randomly chosen points were selected along the high tide line and fixed volumes of sand were extracted in each point. The sampling was done at dawn to avoid interference from visitors.
Description of data collection	Quantification of microplastics was done by extracting them from the sand samples, combining flotation and filtration techniques. The classification of types of microplastics and colour was done by direct visual inspection, allowing to identify fibers, pellets, rigid and semirigid fragments, spheres (ammunition for toys), foams, and films. FTIR spectrophotometry was used to identify the prevalent raw material in each plastic particle. Results for quantification of microplastics in different beaches are presented as the median of ten samples, while the analysis of colour, types of microplastic and raw material are shown in percentage.
Data source location	Institution: Universidad Autónoma de Baja California / Universidad Autónoma Metropolitana City/Town/Region: 35 sandy beaches along the Mexican coast Country: Mexico Latitude and longitude for collected samples: presented in the paper
Data accessibility	Raw data can be accessed at https://data.mendeley.com/datasets/hwr5h6r79n/2
Related research article	Microplastics in Mexican beaches. Alvarez-Zeferino, J. C., Ojeda-Benítez, S., Cruz-Salas, A. A., Martínez-Salvador, C., & Vázquez-Morillas, A. (2020). Microplastics in Mexican beaches. Resources, Conservation and Recycling. 155 (2020) 104633 DOI:10.1016/j.resconrec.2019.104633

**Value of the Data**

• Data, obtained through a consistent methodology, about the presence of microplastics in Mexican beaches are presented
• Results can be used to analyze the origin and effects of microplastics, as well as to compare to other regions or countries
• A complete set of information regarding specific characteristics of microplastics and a detailed explanation of the methods can be used to replicate this kind of study
• It allows comparison between marine regions
• It includes the characterization of all the microplastic particles found in the sampling

## 1 Data Description

The dataset describes the presence of microplastics in 35 sandy beaches along the Mexican coast. Table 1 shows the location of the beaches selected for the study, referring them to the oceans and marine regions that surround Mexico. The map showing the location of the selected points is presented in the article Microplastics in Mexican beaches [1].

**Table 1:** Location of the beaches included in this research

Ocean	Marine Region	State	Beach	ID	Coordinates (Decimal degrees) West	Coordinates (Decimal degrees) North
Atlantic	Gulf of Mexico	Tamaulipas	Miramar	GM 1	-97.798	22.282
		Veracruz	Tamiahua	GM 2	-97.420	21.290
			Tuxpan	GM 3	-97.307	20.975
			Tecolutla	GM 4	-97.007	20.481
			Chachalacas	GM 5	-96.321	19.431
			Sontecomapan	GM 6	-94.993	18.558
		Tabasco	Caracol	GM 7	-93.222	18.437
			Brujas	GM 8	-93.103	18.443
		Campeche	Acapulquito	GM 9	-90.707	19.542
		Yucatán	Progreso	GM 10	-89.635	21.291
	Caribbean Sea	Quintana Roo	Holbox	CS 1	-87.378	21.527
			Del Carmen	CS 2	-87.066	20.630
			Mahahual	CS3	-87.707	18.717
Pacific	Tropical Pacific	Oaxaca	Zipolite	TP 1	-96.518	15.663
			San Agustinillo	TP 2	-96.545	15.665
		Guerrero	Troncones	TP 3	-101.749	17.811
			Ventura	TP 4	-98.916	16.539
		Michoacán	Colola	TP5	-103.346	18.270
		Colima	Miramar	TP6	-104.387	19.118
	Gulf of California	Nayarit	Punta Pelicanos	GC 1	-105.373	20.751
			El Borrego	GC 2	-105.279	21.525
			Novillero	GC 3	-105.685	22.375
		Sinaloa	Las Glorias	GC 4	-108.533	25.298
			Brujitas	GC 5	-106.488	23.301
		Sonora	El Cochorit	GC 6	-110.775	27.917
			Bahía de Kino	GC 7	-112.030	28.860
			Puerto Peñasco	GC 8	-113.541	31.297
			Golfo de Santa Clara	GC 9	-114.495	31.680
		Baja California	El Paraíso	GC 10	-116.926	32.233
			San Felipe	GC 11	-114.833	31.022
		Baja California Sur	El Burro	GC12	-111.907	26.729
			Balandra	GC 13	-110.349	24.142
	Pacific Northwest	Baja California	Ensenada	PN 1	-116.609	31.828
		Baja California Sur	Cerritos	PN 2	-110.174	23.327
			Medano	PN 3	-109.895	22.895

Table 2 shows the mean and median values for the quantification of microplastics in ten random sampling places (ten samples per beach) along the high tide line. The mass of sand is presented on a wet and dry basis. Microplastics are quantified by mass and number of pieces. The number of pieces accounts for all the microplastics found on each beach. Mean and median values are presented for the ten sampling points on each beach.

**Table 2:** Quantification of microplastics (MP) in sampled Mexican beaches (average of ten samples)

Beach ID	Beach class ^1^	Wet sand mass (kg)	Dry sand mass (kg)	MP mass (mg)	# MP (pieces)	Mean (#MP/m2) ± STD DEV	Median (#MP/m2)
GM 1	U(O)	2.2 ± 0.2	1.9 ± 0.2	27.0	17	59.9 ± 44.1	70.4
GM 2	R	2.4 ± 0.1	2.0 ± 0.1	18.0	10	35.2 ± 37.1	35.2
GM 3	U(D)	2.3 ± 0.1	1.8 ± 0.1	45.0	19	66.9 ± 53.7	70.4
GM 4	U(D)	2.2 ± 0.2	1.8 ± 0.2	32.0	18	63.4 ± 77.5	17.6
GM 5	U(D)	2.4 ± 0.2	1.9 ± 0.1	24.0	15	52.8 ± 41.5	70.4
GM 6	R	2.7 ± 0.2	2.4 ± 0.2	559.1	34	119.7 ± 99.9	123.2
GM 7	R	2.4 ± 0.1	2.0 ± 0.1	18.0	12	42.3 ± 36.4	35.2
GM 8	R	-	-	-	-	-	-
GM 9	U(D)	2.2 ± 0.3	1.7 ± 0.2	25.0	15	52.8 ± 47.7	52.8
GM 10	U(D)	2.1 ± 0.1	1.8 ± 0.1	30.0	24	84.5 ± 55.6	70.4
CS 1	U(D)	2.7 ± 0.2	2.1 ± 0.1	193.5	19	66.9 ± 63.1	52.8
CS 2	U(O)	2.3 ± 0.1	2.0 ± 0.1	36.0	32	112.7 ± 82.7	88.0
CS 3	U(D)	-	-	-	-	-	-
TP 1	U(D)	2.2 ± 0.2	1.8 ± 0.1	19.0	13	45.8 ± 33.4	35.2
TP 2	U(D)	2.3 ± 0.2	2.2 ± 0.2	84.0	38	133.8 ± 49.2	158.5
TP 3	U(D)	2.3 ± 0.1	2.0 ± 0.1	37.0	37	130.3 ± 57.6	140.8
TP 4	U(D)	2.1 ± 0.1	1.8 ± 0.1	26.0	12	42.3 ± 32.4	35.2
TP5	R	2.3 ± 0.1	2.0 ± 0.1	43.0	29	102.1 ± 73.2	123.2
TP6	U(D)	2.4 ± 0.1	2.0 ± 0.1	51.0	36	126.8 ± 47.5	105.6
GC 1	U(D)	2.2 ± 0.2	1.8 ± 0.2	69.0	55	193.7 ± 76.5	193.7
GC 2	U(D)	2.4 ± 0.2	2.1 ± 0.1	26.0	23	81.0 ± 52.6	70.4
GC 3	U(D)	2.3 ± 0.1	2.0 ± 0.1	44.0	33	116.2 ± 33.4	105.6
GC 4	R	2.3 ± 0.1	2.0 ± 0.1	12.0	11	38.7 ± 38.8	35.2
GC 5*	U(D)	2.1 ± 0.2	1.9 ± 0.2	164.0	155	545.8 ± 264.1	475.4
GC 6	R	2.2 ± 0.2	1.8 ± 0.1	42.0	32	112.7 ± 61.7	105.6
GC 7	U(D)	2.2 ± 0.2	1.7 ± 0.2	60.0	29	102.1 ± 71.3	88.0
GC 8	U(O)	2.2 ± 0.2	1.9 ± 0.2	30.0	16	56.3 ± 37.9	70.4
GC 9	U(D)	2.4 ± 0.1	2.0 ± 0.1	34.0	22	77.5 ± 57.0	70.4
GC 10	U(D)	2.4 ± 0.2	1.9 ± 0.1	54.0	27	95.1 ± 57.6	88.0
GC 11	U(D)	2.3 ± 0.2	2.0 ± 0.1	52.0	30	105.6 ± 83.0	70.4
GC12	R	2.3 ± 0.1	1.9 ± 0.1	17.0	11	38.7 ± 38.8	35.2
GC 13	R	2.4 ± 0.1	2.0 ± 0.1	21.0	9	31.7 ± 30.8	35.2
PN 1	U(O)	2.3 ± 0.2	1.9 ± 0.2	41.0	17	59.9 ± 47.1	52.8
PN 2	U(D)	2.3 ± 0.2	2.0 ± 0.2	33.0	15	52.8 ± 38.0	52.8
PN 3	U(O)	2.4 ± 0.2	2.0 ± 0.1	38.0	24	84.5 ± 64.7	70.4

The type of beach is mainly determined by the level of human activities and development in the area. R: Rural; U(D): Urban and developed; U(O): Urban and overdeveloped.

(-) Not sampled due to extreme weather events and/or presence of S*argassum* seaweed

* An extreme weather event took place in the area before the sampling^.^

Table 3 presents the classification of the different types of microplastics found on each beach. The total number of pieces was classified.

**Table 3:** Types of microplastics found in each beach

Beach ID	Film	Rigid and semirigid	Fibers	Pellet	Ammunition for toys (spheres)	Foam	Total
GM1	2	10	1	1	2	1	17
GM2	1	6	0	1	0	2	10
GM3	2	11	1	3	1	1	19
GM4	0	11	1	2	4	0	18
GM5	1	9	2	2	1	0	15
GM6	6	22	2	4	0	0	34
GM7	1	7	1	1	2	0	12
GM8	-	-	-	-	-	-	-
GM9	2	8	1	1	1	2	15
GM10	2	19	0	0	0	3	24
CS1	2	17	0	0	0	0	19
CS2	3	15	11	0	0	3	32
CS3	-	-	-	-	-	-	-
TP1	1	9	1	0	0	2	13
TP2	1	7	24	0	3	3	38
TP3	1	10	0	0	0	1	12
TP4	2	24	4	1	0	6	37
TP5	4	15	6	2	0	2	29
TP6	4	15	4	4	2	7	36
GC1	4	31	12	3	2	3	55
GC2	0	20	2	1	0	0	23
GC3	3	21	4	1	0	4	33
GC4	2	7	0	0	0	2	11
GC5*	17	74	9	6	4	45	155
GC6	3	20	2	2	0	5	32
GC7	1	20	5	0	0	3	29
GC8	1	7	3	0	1	4	16
GC9	2	12	4	0	0	4	22
GC10	0	16	7	0	0	4	27
GC11	0	18	9	0	0	3	30
GC12	0	10	1	0	0	0	11
GC13	2	6	1	0	0	0	9
PN1	2	11	0	1	1	2	17
PN2	2	5	1	2	0	5	15
PN3	3	15	6	0	0	0	24

(-) Not sampled due to extreme weather events and/or presence of S*argassum* seaweed

* An extreme weather event took place in the area before the sampling

Table 4 presents the classification of microplastics by colour. All the microplastics found in each beach were classified.

**Table 4:** Classification of microplastics found in Mexican beaches by colour

Beach ID	Blue	Green	Yellow	Red	Orange	Clear	White	Black	Grey	Purple	Total
GM1	4	2	2	0	0	0	3	6	0	0	17
GM2	2	2	2	1	0	1	2	0	0	0	10
GM3	3	4	3	1	0	3	5	0	0	0	19
GM4	4	5	4	1	0	1	2	1	0	0	18
GM5	3	4	3	1	0	1	3	0	0	0	15
GM6	1	1	0	0	0	7	22	2	1	0	34
GM7	3	3	1	1	1	2	1	0	0	0	12
GM8	-	-	-	-	-	-	-	-	-	-	-
GM9	2	3	2	1	1	2	3	1	0	0	15
GM10	3	4	2	3	0	4	4	1	3	0	24
CS1	1	3	0	1	0	5	8	0	1	0	19
CS2	5	5	6	2	0	3	9	1	1	0	32
CS3	-	-	-	-	-	-	-	-	-	-	-
TP1	3	1	4	0	0	2	3	0	0	0	13
TP2	20	0	0	1	0	4	12	1	0	0	38
TP3	2	3	1	2	0	2	1	1	0	0	12
TP4	6	10	5	1	2	2	8	2	1	0	37
TP5	6	9	4	1	0	4	3	1	1	0	29
TP6	7	4	7	3	0	5	7	2	0	1	36
GC1	13	4	6	3	3	15	4	4	2	1	55
GC2	1	6	2	1	1	4	7	0	1	0	23
GC3	6	5	5	3	1	3	8	1	0	1	33
GC4	2	2	1	1	0	0	4	0	0	1	11
GC5*	20	23	30	15	2	12	37	0	10	6	155
GC6	4	8	6	3	0	4	6	1	0	0	32
GC7	4	3	7	2	1	4	7	1	0	0	29
GC8	3	3	4	2	1	0	2	1	0	0	16
GC9	4	3	5	1	0	2	5	0	1	1	22
GC10	3	5	8	2	0	2	4	1	1	1	27
GC11	6	4	3	2	1	3	10	0	0	1	30
GC12	2	3	2	1	0	0	3	0	0	0	11
GC13	1	3	0	0	0	1	3	0	1	0	9
PN1	3	3	2	1	3	1	3	0	0	1	17
PN2	2	4	5	0	0	2	2	0	0	0	15
PN3	2	5	7	1	0	3	6	0	0	0	24

(-) Not sampled due to extreme weather events and/or presence of S*argassum* seaweed.

* An extreme weather event took place in the area before the sampling

Table 5 shows the classification of microplastics by type of polymer. All the microplastics found in the beaches, bigger than 2 mm, were classified.

**Table 5:** Classification of microplastics by polymer

Beach ID	PE	PP	PS	PU	PVC	PET	Nylon	PC	Others	Total
GM1	8	1	1	0	0	0	0	0	0	10
GM2	6	2	1	1	0	0	0	0	0	10
GM3	11	3	0	1	0	0	0	0	0	15
GM4	8	3	1	0	0	0	0	0	0	12
GM5	10	2	0	0	1	0	0	0	0	13
GM6	10	5	18	0	1	0	0	0	0	34
GM7	7	2	0	0	0	0	0	0	0	9
GM8	-	-	-	-	-	-	-	-	-	-
GM9	6	2	0	2	1	0	0	1	0	12
GM10	9	4	0	0	2	0	0	0	0	15
CS1	4	3	7	0	1	0	0	0	1	16
CS2	13	11	1	2	0	0	0	0	0	27
CS3	-	-	-	-	-	-	-	-	-	-
TP1	5	2	1	1	0	0	1	0	0	10
TP2	1	2	11	0	0	0	0	0	1	15
TP3	4	2	0	0	1	0	0	1	0	8
TP4	17	6	6	0	2	0	0	1	0	32
TP5	9	7	2	0	1	0	0	0	0	19
TP6	15	8	4	3	0	0	0	0	0	30
GC1	22	7	2	1	1	0	4	1	0	38
GC2	14	4	0	0	0	0	0	1	0	19
GC3	15	7	2	2	0	0	0	1	0	27
GC4	7	2	1	1	0	0	0	0	0	11
GC5*	60	19	28	17	2	0	0	0	0	126
GC6	16	4	4	1	3	0	0	0	0	28
GC7	14	9	1	0	0	0	0	0	0	24
GC8	6	3	2	2	0	0	0	1	0	14
GC9	12	5	0	1	0	0	0	0	0	18
GC10	9	6	2	2	0	0	0	0	0	19
GC11	12	9	1	0	1	0	2	0	0	25
GC12	6	2	0	0	0	0	0	1	0	9
GC13	4	2	0	0	0	0	1	0	0	7
PN1	9	2	0	2	0	0	0	0	0	13
PN2	4	3	0	4	0	0	0	0	0	11
PN3	15	7	0	0	1	0	0	0	0	23

PE: polyethylene, PP: polypropylene, PS: polystyrene, PU: polyurethane, PVC: polyvinyl chloride, PET: polyethylene terephthalate, PC: polycarbonate

(-) Not sampled due to extreme weather events and/or presence of S*argassum* seaweed

* An extreme weather event took place in the area before the sampling

## 2 Experimental Design, Materials and Methods

### 2.1 Sampling of microplastics

In each beach ten randomly selected points along the high tide line were selected for sampling. The app Aleatorio UX® was used to select them [2], [3]. A polyvinyl chloride cylinder (0.19 m diameter, 0.05 m depth) was pushed into the sand until its upper border reached the surface of the sand. A flat stainless steel sheet was introduced under the cylinder to remove the sand. Each sample was kept in a sealed bag for its analysis in the laboratory. To avoid cross-contamination produced by the sampler, before each sampling, the sampler´s condition was evaluated to ensure no burr was present. When MPs with the same characteristics of the PVC sampler (white and rigid) were seen within the samples, they were removed and separately inspected, to ensure they were not produced by fragmentation of the sampler. Altought this could no guarantee that cross contamination did not take place, the results shown in the Supplementary material leads to assume that possible abrasion of the sampler did not produce a significative effect in the overall results.

### 2.2 Extraction of microplastics

Each sample was weighed (OHAUS model AP110 with ±0.0001 g precision) before and after drying in at over at 105°C for 24 h, to report the results on a dry basis. Microplastics were extracted by flotation, submerging the samples in a CaCl2 solution (ρ ≈ 1.6 g/ml). Plastic particles floated in this media and were extracted with tweezers. This method allowed for the extraction of microplastics in the 0.5–5.0 mm range.

### 2.3 Elimination of false positives

To avoid false positives, the extracted microplastics were dried in an oven (60°C for two hours) and submerged in an acidic solution (HCL 0.5 N), that allowed the identification of shells by the formation of bubbles. Then the microplastics were introduced in an oxidant solution (30% v/v H_2_O_2_), to oxidize the organic matter [4].

### 2.4 Quantification and classification of microplastics

All the microplastics were quantified, mean and median values were calculated for the ten samples of each beach. The concentration of microplastics was calculated as the number of pieces by surface area for each sampling point and then mean and median values were calculated.

Classification by type and colour was done by direct visual inspection. Chemical composition was analyzed in a Spectrum Two FT-IRL-160000F Perkin Elmer Fourier Transform Infrared Spectroscopy (FTIR-ATR). Only samples bigger than 2 mm could be analyzed in the equipment due to the precision of the ATR. The measurements were done in the 400–4000 cm-1 range, with a resolution of 4 cm-1 and 32 scans. The resulting spectra were compared to the library included in the FTIR.

## CRediT Author Statement

**Juan Carlos Alvarez-Zeferino:** Concepturalization, Formal Analysis, Investigation, Writing – Original Draft. **Sara Ojeda-Benítez:** Supervision, Writing – Review & Editing. **Arely Areanely Cruz-Salas:** Methodology, Validation, Writing – Original Draft. **Carolina Martínez-Salvador:** Writing - Review And Editing, Visualization. **Alethia Vázquez Morillas:** Conceptualization, Supervision.

## Declaration of Competing Interest

The authors declare that they have no known competing financial interests or personal relationships which have, or could be perceived to have, influenced the work reported in this article.

## Appendix Supplementary materials

Image, application 1
