# Supplementary material for: Dataset of quantification and classification of microplastics in Mexican sandy beaches
Source: Data Brief. 2020 Oct 31;33:106473. doi: 10.1016/j.dib.2020.106473 (PMC7649470; doi:10.1016/j.dib.2020.106473)
Supplement: Supplementary file 1 [file mmc1.pdf]

Resources, Conservation & Recycling 155 (2020) 104633

Contents lists available at ScienceDirect

Resources, Conservation & Recycling

journal homepage: www.elsevier.com/locate/resconrec

Full length article

Microplastics in Mexican beaches

Juan Carlos Alvarez-Zeferino[a], Sara Ojeda-Benítez[a], Arely Areanely Cruz-Salas[b], Carolina Martínez-Salvador[c], Alethia Vázquez-Morillas[b,*]

[a] Universidad Autónoma de Baja California – Instituto de Ingeniería, UABC, Boulevard Benito Juárez y Calle de la Normal S/N, Col. Insurgentes Este, C.P. 21280, Mexicali, Baja California, Mexico
[b] Universidad Autónoma Metropolitana - Azcapotzalco, Av. San Pablo, No. 180 Col. Reynosa-Tamaulipas, C.P. 02200, Ciudad de Mexico, Mexico
[c] MSc on Water Sciences and Engineering, IHE-UNESCO, Westvest 7, 2611 AX, Delft, the Netherlands

ARTICLE INFO

Keywords:
Microplastic pollution
Marine waste
Gulf of California
Gulf of Mexico
Caribbean Sea

ABSTRACT

Mexican sandy beaches are a natural resource that provides environmental services and contribute to the economic growth of the country. However, due to mismanagement of solid waste, discharges of wastewaters and other land and marine activities, they can be polluted by microplastics. This research aimed to quantify and classify the microplastics found in Mexican beaches. Samples were taken in 33 beaches, along the five marine regions of the country, following a systematic method that allows comparison of results. Microplastics concentration varied from 31.7–545.8 M P/m^2, with high variance coefficients (28.7–122.3 %). The Gulf of California was the region that showed a higher mean concentration. Presence of microplastics was higher in urban-overdeveloped beaches and increased with the occurrence of extreme weather events. Prevention of pollution of beaches by microplastics would allow to preserve the natural resources of the country, as well as to recover valuable materials that can be reinserted in production cycles.

1. Introduction

Mexico, located in North and Central America, is a country with great biodiversity and rich in natural resources. Mexican beaches are famous by its warm and colorful waters, soft sand and mild weather, attracting millions of visitors every year. In 2016 tourism accounted for 8.5 % of the GDP of the country (OECD, 2017), which received 41 million visitors (SECTUR, 2018). Mexican beaches face challenges regarding their preservation and the sustainability of the touristic and economic activities that take place in them. Lack of regulations or reinforcement of existing laws, deficient management of waste and discharge of wastewater cause pollution of the water and the shore, regardless of the “Clean beach” programme promoted by the Federal Authorities.

One of the environmental problems faced by these ecosystems is the presence of solid waste. More than 80 % of the waste in marine ecosystems is made up of plastics, generated mainly from land-based sources (Fossi et al., 2016). In the ocean, plastics can be transported by the currents, giving plastic pollution a global reach. It has been estimated that between 4.8–12.7 million of metric tons of plastic waste were discarded into to the ocean each year, accounting from 1.7 to 4.6 % of the annual global production of plastics, and taking into account the mismanagement practices of 192 countries. For Mexico, contribution to plastic input into the ocean has been calculated as 0.01 to 0.25 million of metric tons (Jambeck, et al., 2015). A specific category among marine plastic waste is the group of particles known as microplastics, which measure < 5 mm (Liu et al., 2019). This range has been defined by the availability of sampling techniques, detection limits and the probability that the particles are ingested by the aquatic fauna (GESAMP, 2015).

Microplastics can be primary when they were originally produced on a microplastic size, or secondary, when they are the result of the degradation of bigger plastic items. The main sources of primary microplastics are discharges of wastewater, the presence of microbeads in personal care products and leaks of pellets during transportation or manufacturing (Anderson et al., 2016; Conkle et al., 2018; Kalčíková et al., 2017; Veerasingam et al., 2016a). Secondary microplastics, on the other hand, proceed from the mismanaged waste in coastal cities, beaches and marine activities, such as fishing (Deshpande et al., 2019), and leaks in global plastic waste trade networks (Wang et al., 2020). Plastics stranded on beaches break down by the effect of the UV radiation, oxidation, friction and other concurrent factors that promote degradation (Brandon et al., 2016; Siegfried et al., 2017; Tsiota et al., 2018).

* Corresponding author.
E-mail address: alethia@azc.uam.mx (A. Vázquez-Morillas).

https://doi.org/10.1016/j.resconrec.2019.104633
Received 30 March 2019; Received in revised form 3 December 2019; Accepted 3 December 2019
0921-3449/ © 2019 Published by Elsevier B.V.

The presence of microplastics in marine ecosystems presents a greater risk than macroplastics (Walker and Xanthos, 2018). Microplastics can be ingested by marine fauna (Derraik, 2002; Germanov et al., 2018; Ogonowski et al., 2018). In the other hand, due to their hydrophobicity, they can adsorb pollutants found in the water (Frias et al., 2010; Holmes et al., 2012; Kedzierski et al., 2018; Wilkinson et al., 2017). When microplastics are ingested by different species, those adsorbed pollutants and additives used on some plastics can leach and enter the trophic food chain (Fossi et al., 2016; Martellini et al., 2018).

Extensive research has been done to analyze the presence, distribution, and characteristics of microplastics in beaches around the world. Only in 2018, using the keywords "microplastics" + "sand" 67 new research papers related were found in ScienceDirect®. It has been proposed that the presence of microplastic on marine waters on specific areas is the result of marine currents, while their presence in beaches is related with the human activities that take place in them (Shim et al., 2018), although some others, like Martellini et al. (2018) analyzed the presence of microplastics in 16 beaches in the Mediterranean Sea and found no correlation between touristic activities and presence of microplastics, attributing them to sedimentation due to wastewater from land activities and transport by rivers (Kataoka et al., 2019).

Regardless of the natural and economic relevance of Mexican beaches, there is limited information regarding their pollution by microplastics. The research has focused on the presence of microplastic in marine waters, specifically in the Mar de Cortés (Fossi et al., 2016) and the Southern portion of the Gulf of California (Fossi et al., 2017). The first one reported that concentration of microplastics were four times lower than in the Mediterranean Sea, while the second one analyzed possible correlations between ingestion of microplastics and presence of persistent organic pollutants in the adipose tissues of whales. The study of the Atoyac basin, in the central region of the country, confirmed the influence of wastewater discharges and urbanization on the presence of microplastics (Shruti et al., 2019), a fact that has been reported before (Wen et al., 2018).

The knowledge about the quantities and characteristics of the microplastics that were found will help to estimate the extension of the problem, as well as to identify critical regions and factors which require immediate attention (Ryberg et al., 2019). This research aims to provide information about the presence of microplastics in Mexican beaches, by a systematic sampling campaign in beaches of the five marine regions of the country.

2. Material and methods

2.1. Selection of beaches

The Mexican shoreline has 11,122 km of length. Seventeen of the 32 states of the country have direct access to the sea (CIMARES, 2010). The national coast has been divided into five marine regions: Pacific Northwestern, Gulf of California, Tropical Pacific, Caribbean Sea and Gulf of Mexico (Arriaga-Cabrera et al., 1998). For this research, at least three beaches of each region were selected. The selection was based on the following criteria: sandy beaches, with touristic activity, a minimal length of 100 m, at least at one kilometer from docks, easy access, and good safety conditions. Selected beaches were classified as proposed by Barbosa de Araújo and da Costa (2008) in three categories, shown in Table 1.

2.2. Sampling of microplastics

Field sampling activities took place from April to July 2018. Samples were taken along the high tide line, where sediments and microplastics tend to accumulate (Cole et al., 2011; Lavers and Bond, 2017). A 100 m segment was chosen and marked with a rope, which was extended parallel to the high tide line. Ten sampling points were randomly selected along the line, using the app Aleatorio UX®, which produced integer numbers between one and 527 (527 = 100 m/0.19 m; 0.19 m is the diameter of the sampler. The position of each point was located in the rope, and then projected through a perpendicular line to the high tide line. Samples were taken with a polyvinyl chloride cylinder (19 cm diameter and 5 cm depth). It was sunk until its upper edge reached the surface of the sand. Then, the sampler containing a fixed volume of sand was extracted by inserting a stainless-steel sheet under it. The samples were wrapped in foil and put into bags to be transported to the lab.

2.3. Extraction and classification of microplastics

The microplastics reported in this research are in the range of 0.5–5 mm. Each sample was weighed, dried in an oven (105 °C, 24 h) and weighed again. It was considered that the drying temperature would not affect the structure or composition of microplastics, as common plastics have a melting point above 115 °C (Braun, 2013). Microplastics were extracted by flotation. The dried sand was passed through a #16 mesh sieve (1.13 mm). The materials retained in the mesh were submerged in a $CaCl_2$ solution with $\rho \approx 1.6$ g/ml, as suggested by Kedzierski et al. (2016). The solution was mixed for one minute and allowed to settle for one minute. Floating particles were extracted with stainless steel tweezers, washed with distilled water and dried for two hours at 60 °C. Fragments of shells and biogenic wastes can be mistaken as microplastics. To avoid false positives, the extracted particles were put in contact first with an acidic solution (HCl 0.5 N) to eliminate shells and then with an oxidant solution (30 % v/v H_2O_2), to identify and remove organic matter (Free et al., 2014). To avoid contamination of the sample glass and metal tools and recipients were used, to avoid direct contact of the samples with plastic surfaces.

The microplastics found in each sampling point were counted and their total mass was weighted. Then they were classified by type (fibers, pellets, rigid and semirigid fragments, foams and films), color and chemical composition. This last parameter was analyzed in all the microplastics in the $2-5$ mm range by Fourier Transform infrared spectroscopy (FTIR), in a Spectrum Two FT-IRL-160000A, 160,000 F Perkin Elmer. The measurements were done in the 400–4000 cm-1 range, with a resolution of 4 cm-1 and 32 scans. FTIR is one of the main techniques used for identification and prediction of the degradation degree for polymers (Alassali et al., 2020).

2.4. Analysis of results

The concentration of microplastics was calculated as the number of pieces by the surface area, for each sampling point (MP/m^2). Mean and

Table 1
Categories for the classification of beaches.

Type of beach	Characteristics
Rural	Forested area and/or palm trees; low interaction between human activities and the environment; scarcity of facilities, infrastructure, and trade
Urban	Presence of holiday homes, medium level of interaction between human activities and the environment; low presence of facilities, infrastructure, and commercial activity.
Urban overdeveloped	Continuous constructions, some with more than five floors; high level of interaction between human activities and the environment, great variety of uses; numerous facilities, infrastructure, and commercial activities.

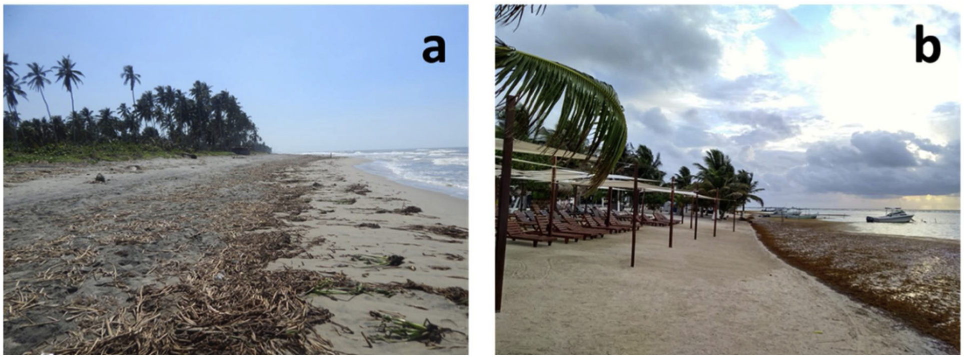

Fig. 1. Biogenic marine waste in beaches in a) the Gulf of Mexico, and b) the Caribbean Sea.

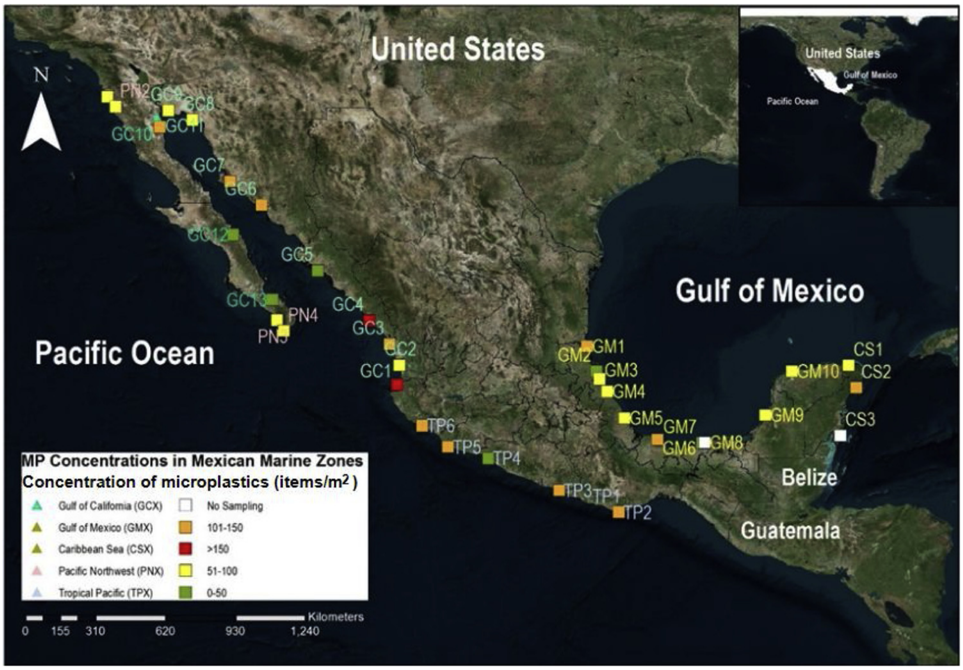

Fig. 2. Concentration of microplastics in Mexican beaches.

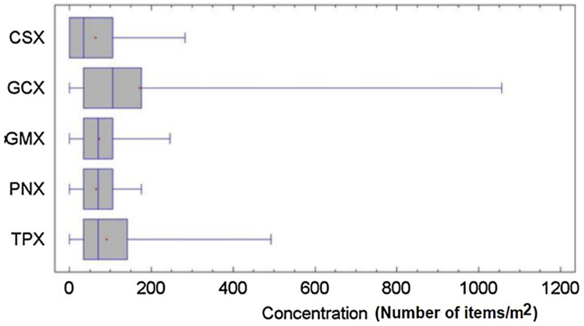

Fig. 3. Comparison of MP concentration in different marine regions (GCX: Gulf of California, GMX: Gulf of Mexico, CSX: Caribbean Sea, PNX: Pacific Northwest, TPX: Tropical Pacific). The graph shows the minimum and maximum values, lower and upper quartile. The symbol "+" shows the mean value, the vertical line inside the boxes is the median.

median values, variance (%) and limits were calculated for each beach. Differences between marine regions were analyzed with STATGRAPHICS®, using a non-parametric Mood test, to assess if there were significant differences between the median values, with a 95 % confidence interval. Median, instead of mean values, were used considering that the mean (average) can be more affected by factors such as wind, topographical features and nearby estuaries (Laglbauer et al., 2014).

3. Results and discussion

35 beaches along the Mexican seacoast were visited: 10 in the Gulf of Mexico, three in the Caribbean Sea, six in the Tropical Pacific, 13 in the Gulf of California and three in the Northwest Pacific. However, microplastics were quantified only in 33, due to the presence of biogenic marine waste (seaweed and Sargassum) in beaches located in the Gulf of Mexico and the Caribbean Sea (Fig. 1). This waste prevented the identification of the high tide line. Further research is needed in order to develop sampling methodologies that can be applied under these conditions, as biogenic waste could contain entangled microplastics.

3.1. *Presence of microplastics in Mexican beaches*

Microplastics were found in all the beaches, as shown in Fig. 2. Mean values for different beaches ranged from 31.7–545.8 M P/m^2, with high variance coefficients (28.7–122.3 %). The highest concentration (545.8 M P/m^2) was found in an urban, developed beach located in the state of Sinaloa (GC5), while the lowest one (31.7 M P/m^2) was found in a rural beach of Baja California (GC13). Both beaches

Table 2
Concentrations of microplastics in beaches from different countries.

Country	Number of beaches	Size of microplastics (mm)	Concentrations of microplastics (MP/m^2)	Reference
United States	7	N.S.	5 - 117	Wessel et al., 2016
Brazil	17	N.S.	Summer 12 - 1300<br>Winter 3 - 743	de Carvalho and Baptista Neto 2016
Qatar	8	2	36 - 228	Abayomi et al., 2017
South Korea	3	0.05 – 5	46,334	Kim et al., 2015
South Africa	21	0.065 – 5	688.9 – 3,308	Nel and Froneman, 2015
Uruguay	10	0.3 – 5	25	Lozoya et al., 2016
Perú	4	1 – 2.5	130[1]	Purca and Henostroza, 2017
México	33	1 - 5	31.7 - 545.8<br>Mean: 133	This research

N.S. = Not Specified; 1 = Average hard plastic fragments.

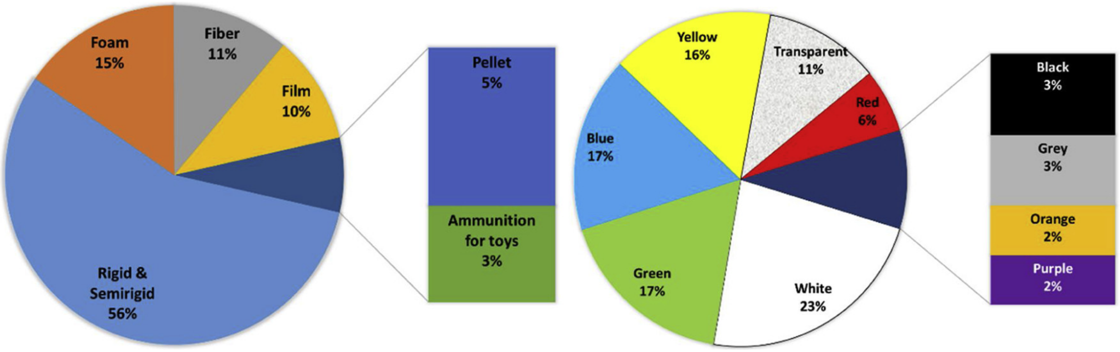

Fig. 4. Types and colors of microplastics found in Mexican beaches (n = 975).

are in the same region, the Gulf of California. However, it should be noticed that five of the studies in the Gulf of California (GC1 to GC5) coincided with Hurricane Bud, a category three tropical cyclone that affected the Northwestern coast of Mexico and the Southwestern coast of the United States (CONAGUA, 2018). This natural phenomenon could have increased the concentration of MP on the beaches; it has been reported that hurricanes and storms can increase the presence of plastics in marine environment, due to leaching of waste from the land, rise in the level of rivers, flooding (Antunes et al., 2018; Cheung et al., 2016) and wind (Browne et al., 2010; Kukulka et al., 2012). In the rainy season the plastics and microplastics dumped in the land, or even in landfills, can leach to rivers, which transport them to the ocean (Eo et al., 2019). Is it common that humid seasons coincide with maximum concentration of microplastics (Veerasingam et al., 2016b).

Nine beaches were classified as rural, 21 as urban-developed and five as urban-overdeveloped. The higher concentrations for the Northwestern Pacific and the Caribbean Sea were found in over-developed beaches. This suggest a correlation between the presence of microplastics and the flow of visitors (Retama et al., 2016; Tiwari et al., 2019). In the Gulf of Mexico, the highest concentration (GM6, 119.7 M P/m^2) was found in a rural beach, however, it is located at 0.5 km of a river mouth, which could be transporting plastic particles from the towns located in the riverside, as proposed previously (Karthik et al., 2018). The shape of the beaches is a factor that also influence the presence of microplastics; in the Tropical Pacific the most polluted beach was a bay (133.8 M P/m^2).

When comparing marine regions (Fig. 3) only the Gulf of California has a statistically different mean, with higher values than the rest (median = 105 M P/m^2). However, this result should not be considered as representative, as this behavior could be related to the presence of hurricane Bud. Further sampling is needed in order to assess if the effect of this type of phenomena is significant, and to assess the concentration of microplastics in the region.

Results of similar researches in other countries are shown in Table 2. The range and mean value for the Mexican beaches are in the same order of magnitude than the reported concentrations, except when it comes to South Africa and South Korea. In the case of South Africa a high proportion (> 50 %) of mismanagement of plastic waste has been reported (Jambeck et al., 2015), while in South Korea the high concentration could be related to the high density of the population in a ratio of 50 km of the coast (UNEP, 2006).

3.2. Characteristics of microplastics

Microplastics can be primary (microbeads, pellets) or secondary, produced by the fragmentation of bigger plastic pieces. This research showed a prevalence of secondary microplastics, with rigid fragments, foams, fibers, and films accounting for 92 % of the found pieces (Fig. 4). The three main categories found in our research (rigid fragments, foams, and fibers) coincide with the results of previously reported results, as shown in Table 3. These secondary microplastics, which have been degraded by sunlight, friction and other physicochemical processes, have a higher risk of adsorption of hydrophobic pollutants, by the increase on their surface area produced by degradation (Vedolin et al., 2019).

Primary microplastics included pellets (5 %) and spherical pieces used as ammunition in compressed air toy guns (3 %). Pellets were found in 57 % of the beaches, with higher concentrations in the Gulf of Mexico. This can be attributed to the presence of the petrochemical industry in the area, which produces polyethylene and polypropylene pellets that can leak during transport and production. Ammunition for toys, on the other hand, was found in 42 % of the beaches. Examples of the microplastics found in this research are shown in Fig. 5.

The shape of microplastics could increase the damaged caused when they are ingested by marine fauna. Pointy rigid fragments can puncture organs and tissues (Ory et al., 2017). The proportions of different shapes of microplastics can be influenced by the land-based activities near the beaches. Estuaries and wastewater discharges could increase the proportion of fibers, while touristic beaches could have higher concentrations of rigid and foamed fragments, produced by the

Table 3
Types of microplastics found on beaches of different countries.

Country/Region	Types of microplastics	Color	Chemical composition	Reference
Europe	Fibers (98.7 %), Particles (0.91 %), Film (0.35 %)	Blue/black (77.5–82.9 %), red (9.3–13.6%)	Polyester (70 %), PP (20 %), PE (10 %)	Lots et al., 2017
India	Fragments (47–50 %), Fibers (24–27 %) and Foam (10–19 %)	N.R.	PE (45.98 %), PP (19.41 %), PS (17.41 %)	Karthik et al., 2018
Mexico	Fibers (91 %), Film (5 %), Spheres (toys) (3 %)	Fibers: black (59 %) and blue (25%). Other colors: purple (7%), red (7%) and green (2%)	Polyacrylamide, Nylon, Polyacrylate	Piñon-Colin et al., 2018
Brazil	Fragments (56 %), Foam (26.7 %), Pellet (9.9 %)	Fibers: blue, red and green. Pellets: translucent	Fibers: PP, Nylon and Polyvinyl alcohol. PS (26.7 %)	de Carvalho and Baptista Neto, 2016
Portugal	Fibers (80.64 %) and Fragments (19.63%)	Fibers: red, green, blue and black. Fragments: blue and green	Fibers: Rayon (81 %). Fragments: PP (19.36 %)	Frias et al., 2016
Colombia	Pellet[1]	White (65.07 %), sand (24.26 %), gray (3.83 %)	PE y PP	Acosta-Coley and Olivero-Verbel, 2015
Peru	Fragments (> 80 %), Foams (8 %), Pellets (2 %)	N.R.	PE (50 %) and PS (20 %)	Purca y Henostroza, 2017
Mexico	Foams (15 %), Fibers (11 %), Rigid and semirigid fragments (56 %), Film fragments (10 %), Pellets and ammunition for toys (8 %)	White (23 %), green (17%), blue (17%), yellow (16%), transparent (11%), red (6%), others (10%9	PE (56 %), PP (21 %), PS (12 %), others (11 %)	This research

N.R. = Not reported; 1 = only pellets were collected; PE = polyethylene, PP = polypropylene, PS = polystyrene.

degradation of disposable cutlery and plastic bags. Further research is needed in order to study the relationship between economic activities, urbanization and presence of specific types of microplastics.

International researches (Table 2) show a prevalence of black, blue, green and red microplastics. On the other hand, Mexican beaches are widely reached by white as the most frequent color, followed by blue and green (Fig. 4). A research done in Portugal reported that white microplastics contained less adsorbed persistent organic pollutants, however, this still must be confirmed by other studies (Frias et al., 2010), in order to assess if there is a correlation between color and adsorption. Color can affect the interactions of microplastics and marine fauna; it has been proposed that dark particles have a higher possibility of being ingested (by accident) and also that some colors could cause microplastics to be mistaken as food (Christian et al., 2018; Ory et al., 2017).

The chemical composition of microplastics can give information regarding the sources of this type of pollutants and possible interactions with other pollutants. The analysis of the chemical composition of microplastics found in Mexican beaches showed the prevalence of polyolefins. Polyethylene (PE) was the main component in 56 % of the particles. High proportions of this material, used for the production of plastic bags, toys and bottles have been reported previously (Karthik et al., 2018; Purca y Henostroza, 2017). Polypropylene (PP), used for disposable containers, straws and water bottle caps, accounted for 21 % and expanded polystyrene (PS), used in disposable cups and plates, for 12 %. These plastics have been reported as prevalent in other studies (Table 2). However, it must be noticed that their presence could also be related to their physical properties; while other plastics like polyethylene terephthalate and vinyl polychloride have a higher density than water and therefore they sink PE, PP and PS tend to float, and have a higher possibility of being transported by rivers and currents.

Limitations of this study include the lack of technical capability to identify and characteriza microplastics smaller than 0.5 mm, the exclusion of beaches whose sediments are different than sand, and the depth used for the sampling (0.05 m). This factors need to be taken into account when comparing the results with those obtained in other studies. Mexican coast is much longer and diverse than the specific sites analyzed in this research, so the results can not be considered as representative of the whole country. However, this study allows to identify the order of magnitude of the presence of microplastics, as well as some of their relevant characteristics.

The different marine regions of the country have distinctive sea-based economical activities: tourism in the Caribbean Sea, with high flow of cruises, intensive fishing in the Pacific Ocean and the Gulf of California, and oil extraction and petrochemical industry in the Gulf of Mexico. Regardless this differences, there were no statistically meaningful differences in the concentration of microplastics between the regions, with the exception of the Gulf of California, as discussed before. This suggest a prevalence of land-based activities as a primary source for microplastics that arrive to Mexican beaches. Mexico lacks a national policy focused on the conservation and sustainable use of marine resources, as shown by the absence of this topic in the National Development Plan 2019–2024 (DOF and D.O. de la federación, 2019). The responsibility of the reinforcement of regulations about coastal areas is distributed between the Ministry in charge of the Environment and the Navy, but the care of the beaches is an attribution of local municipalities, which usually lack resources and infrastructure for this job. Only two regulatory instruments have been developed by the authorities at the federal level: the first one is the Clean Beaches program, focused in the monitoring of water quality (SEMARNAT, 2019). The results of the program are public, and can affect the affluence of visitors to areas classified as "polluted". In order to prevent the presence of microplastics, the assessment of the presence of solid waste and microplastics, as well as the existence of waste management programs could be included as additional parameters. The risk to affect the local economy could trigger the interest of local authorities in improving

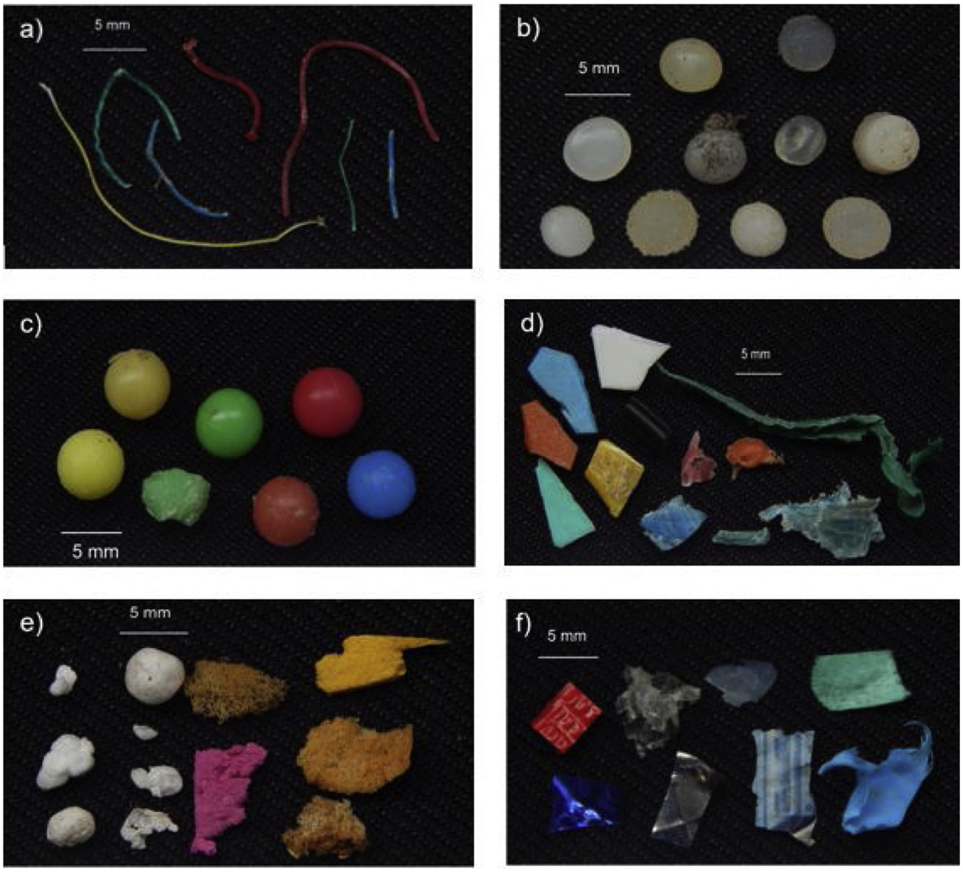

Fig. 5. Examples of microplastics found in this study. a) Fibers, b) Pellets, c) Ammunition for toys, d) Rigid fragments, e) Foams, and f) Film fragments.

waste management in beaches.

The second instrument is a voluntary certification program that assess the sustainability of beaches (de energía Sener, 2006). The standard distinguishes between recreational and conservation beaches, defining specific quality parameters for different environmental factors, including waste management. The impact of the program is limited; only nine beaches are recognized as “Sustainable clean beach” by the authorities. Clearly, in order to increase the benefits derived from this program, there is a need for incentives and support for the local governments; the federal authorities should design promotion schemes trough capacitation, funding and mentoring. Microplastics are not included now in the standard, they should be introduced as an environmental quality indicator.

4. Conclusion

This research showed clear evidence of the presence of microplastics in Mexican sandy beaches (31.7–545.8 M P/m^2), in similar concentrations to those found in other parts of the world. The use of a standardized methodology allowed to compare results between the different beaches and marine regions. The highest concentrations of microplastics, found in the Gulf of California, coincided with the arrival of hurricane Bud. Due to its location and size, Mexico faces frequent extreme weather events. Specific studies are needed in order to better understand how these events affect the distribution of microplastics and other pollutants in the environment.

Tourism is a basic activity for the national economy and the sound development in coastal areas of Mexico. However, lack of proactive waste management plans, which prevent pollution by solid waste, hinders the attractive of the beaches and could lead to public health problems. An equilibrium must be pursued between increasing the number of visitors and touristic offer and preserve the natural resources. Sound waste management policies, designed specifically for touristic beaches, must be implemented in an orderly and articulated way. Special attention must be paid to recreational activities done in the beach; as it can be seen by the presence of toy’s ammunition, they can become a direct source of primary microplastics.

It must be noticed that thermoplastics accounted for the majority on microplastics found in this study. Plastics such as polyethylene, polypropylene and polyethylene terephthalate, are highly recyclable. Proper management of this kind of waste, which usually comes from sale and distribution of food and beverages would prevent the formation of microplastics, as well as to promote recycling of materials that could be reintroduced to productive activities.

Mexican beaches are a source of natural, economic and spiritual richness. Further collaboration by authorities, academics, industry, and people is required in order to preserve them and sustainably obtain all the benefits that they provide.

Declaration of Competing Interest

The authors declare that there are no conflicts of interest.

References

Abayomi, O.A., Range, P., Al-Ghouti, M.A., Obbard, J.P., Almeer, S.H., Ben-Hamadou, R., 2017. Microplastics in coastal environments of the Arabian Gulf. Mar. Pollut. Bull. 124, 181–188. https://doi.org/10.1016/j.marpolbul.2017.07.011.

Acosta-coley, I., Olivero-verbel, J., 2015. Microplastic resin pellets on an urban tropical beach in Colombia. Environ. Monit Assess 187, 435. https://doi.org/10.1007/s10661-015-4602-7.

Alassali, A., Picuno, C., Bébien, T., Fiore, S., Kuchta, K., 2020. Validation of near infrared spectroscopy as an age-prediction method for plastics. Resour. Conserv. Recycl. 154. https://doi.org/10.1016/j.resconrec.2019.104555.

Anderson, A.G., Grose, J., Pahl, S., Thompson, R.C., Wyles, K.J., 2016. Microplastics in personal care products: exploring perceptions of environmentalists, beauticians and

students. Mar. Pollut. Bull. 113, 454–460. https://doi.org/10.1016/j.marpolbul.2016.10.048.
Antunes, J., Frias, J., Sobral, P., 2018. Microplastics on the Portuguese coast. Mar. Pollut. Bull. 131, 294–302. https://doi.org/10.1016/J.MARPOLBUL.2018.04.025.
Arriaga-Cabrera, L., Vázquez-Domínguez, E., González-Cano, J., Jiménez-Rosenberg, R., Muñoz-López, E., Aguilar-Sierra, V., 1998. Regiones marinas prioritarias de México. México (coords.). http://www.conabio.gob.mx/conocimiento/regionalizacion/doctos/marinas.html.
Barbosa de Araújo, M.C., da Costa, M.F., 2008. Environmental quality indicators for recreational beaches classification. J. Coast. Res. 246, 1439–1449. https://doi.org/10.2112/06-0901.1.
Brandon, J., Goldstein, M., Ohman, M.D., 2016. Long-term aging and degradation of microplastic particles: comparing in situ oceanic and experimental weathering patterns. Mar. Pollut. Bull. 110, 299–308. https://doi.org/10.1016/j.marpolbul.2016.06.048.
Braun, D., 2013. Simple Methods for Identification of Plastics, 5a. ed. Hanser publications, Cincinnati, Ohio, Estados Unidos.
Browne, M.A., Galloway, T.S., Thompson, R.C., 2010. Spatial patterns of plastic debris along estuarine shorelines. Environ. Sci. Technol. 44, 3404–3409.
Cheung, P.K., Cheung, L.T.O., Fok, L., 2016. Seasonal variation in the abundance of marine plastic debris in the estuary of a subtropical macro-scale drainage basin in South China. Sci. Total Environ. 562, 658–665. https://doi.org/10.1016/j.scitotenv.2016.04.048.
Christian, N., Gallardo, C., Lenz, M., Thiel, M., 2018. Capture, swallowing, and egestion of microplastics by a planktivorous juvenile fish. Environ. Pollut. 240, 566–573. https://doi.org/10.1016/j.envpol.2018.04.093.
CIMARES - Comisión Intersecretarial para el Manejo Sustentable de Mares y Costas, 2010. Política Nacional de Mares y Costas de México. México.
Cole, M., Lindeque, P., Halsband, C., Galloway, T.S., 2011. Microplastics as contaminants in the marine environment: a review. Mar. Pollut. Bull. 62, 2588–2597. https://doi.org/10.1016/j.marpolbul.2011.09.025.
CONAGUA - Comisión Nacional del Agua, 2018. El huracán Bud de categoría 3 en la escala de Saffir Simpson, se desplaza paralelo a la costa del Pacífico (No. 006–18). Ciudad de México.
Conkle, J.L., Báez Del Valle, C.D., Turner, J.W., 2018. Are we underestimating microplastic contamination in aquatic environments? Environ. Manage. 61, 1–8. https://doi.org/10.1007/s00267-017-0947-8.
de Carvalho, D.G., Baptista Neto, J.A., 2016. Microplastic pollution of the beaches of Guanabara Bay, Southeast Brazil. Ocean Coast. Manage. 128, 10–17. https://doi.org/10.1016/j.ocecoaman.2016.04.009.
Derraik, J.G., 2002. The pollution of the marine environment by plastic debris: a review. Mar. Pollut. Bull. 44, 842–852. https://doi.org/10.1016/S0025-326X(02)00220-5.
Deshpande, P.C., Philis, G., Brattebø, H., Fet, A.M., 2019. Using Material Flow Analysis (MFA) to generate the evidence on plastic waste management from commercial fishing gears in Norway. Resour. Conserv. Recycl. https://doi.org/10.1016/j.rcrx.2019.100024. X 100024.
DOF, D.O. de la federación, 2019. Plan Nacional de Desarrollo 2019-2024. [WWW Document]. URL https://www.dof.gob.mx/nota_detalle.php?codigo=5565599&fecha=12/07/2019, acceso de agosto de 2019 (consultado 8.25.19). .
Eo, S., Hong, S.H., Song, Y.K., Han, G.M., Shim, W.J., 2019. Spatiotemporal distribution and annual load of microplastics in the Nakdong River, South Korea. Water Res. 160, 228–2371. https://doi.org/10.1016/j.watres.2019.05.053.
Fossi, M.C., Baini, M., Panti, C., Galli, M., Jiménez, B., Muñoz-Arnanz, J., Marsili, L., Finoia, M.G., Ramírez-Macías, D., 2017. Are whale sharks exposed to persistent organic pollutants and plastic pollution in the Gulf of California (Mexico)? First ecotoxicological investigation using skin biopsies. Comp. Biochem. Physiol. Part C Toxicol. Pharmacol. 199, 48–58. https://doi.org/10.1016/J.CBPC.2017.03.002.
Fossi, M.C., Marsili, L., Baini, M., Giannetti, M., Coppola, D., Guerranti, C., Caliani, I., Minutoli, R., Lauriano, G., Finoia, M.G., Rubegni, F., Panigada, S., Bérubé, M., Urbán Ramírez, J., Panti, C., 2016. Fin whales and microplastics: the Mediterranean Sea and the Sea of Cortez scenarios. Environ. Pollut. 209, 68–78. https://doi.org/10.1016/J.ENVPOL.2015.11.022.
Free, C.M., Jensen, O.P., Mason, S.A., Eriksen, M., Williamson, N.J., Boldgiv, B., 2014. High-levels of microplastic pollution in a large, remote, mountain lake. Mar. Pollut. Bull. 85, 156–163. https://doi.org/10.1016/j.marpolbul.2014.06.001.
Frias, J.P.G.L., Gago, J., Otero, V., Sobral, P., 2016. Microplastics in coastal sediments from Southern Portuguese shelf waters. Mar. Environ. Res. 114, 24–30. https://doi.org/10.1016/j.marenvres.2015.12.006.
Frias, J.P.G.L., Sobral, P., Ferreira, A.M., 2010. Organic pollutants in microplastics from two beaches of the Portuguese coast. Mar. Pollut. Bull. 60, 1988–1992. https://doi.org/10.1016/j.marpolbul.2010.07.030.
Germanov, E.S., Marshall, A.D., Bejder, L., Fossi, M.C., Loneragan, N.R., 2018. Microplastics: no small problem for filter-feeding megafauna. Trends Ecol. Evol. 33, 227–232. https://doi.org/10.1016/j.tree.2018.01.005.
GESAMP, 2015. Sources, fate and effects of microplastics in the marine environment: a global assessment. In: In: Kershaw, P.J. (Ed.), (IMO/FAO/UNESCO-IOC/UNIDO/WMO/IAEA/UN/UNEP/UNDP Joint Group of Experts on the Scientific Aspects of Marine Environment Protection). Reports Stud 90 GESAMP. https://doi.org/10.13140/RG.2.1.3803.7925. 96 p. 96.
Holmes, L.A., Turner, A., Thompson, R.C., 2012. Adsorption of trace metals to plastic resin pellets in the marine environment. Environ. Pollut. 160, 42–48. https://doi.org/10.1016/j.envpol.2011.08.052.
Jambeck, J.R., Geyer, R., Wilcox, C., Siegler, T.R., Perryman, M., Andrady, A., Narayan, R., Law, K.L., 2015. Plastic waste inputs from land into the ocean. Science 347 (80), 768–771. https://doi.org/10.1126/SCIENCE.1260352.
Kalčíková, G., Alič, B., Skalar, T., Bundschuh, M., Gotvajn, A.Ž., 2017. Wastewater treatment plant effluents as source of cosmetic polyethylene microbeads to freshwater. Chemosphere 188, 25–31. https://doi.org/10.1016/j.chemosphere.2017.08.131.
Karthik, R., Robin, R.S., Purvaja, R., Ganguly, D., Anandavelu, I., Raghuraman, R., Hariharan, G., Ramakrishna, A., Ramesh, R., 2018. Microplastics along the beaches of southeast coast of India. Sci. Total Environ. 645, 1388–1399. https://doi.org/10.1016/J.SCITOTENV.2018.07.242.
Kataoka, T., Nihei, Y., Kudou, K., Hinata, H., 2019. Assessment of the sources and inflow processes of microplastics in the river environments of Japan. Environ. Pollut. 244, 958–965. https://doi.org/10.1016/J.ENVPOL.2018.10.111.
Kedzierski, M., D'Almeida, M., Magueresse, A., Le Grand, A., Duval, H., César, G., Sire, O., Bruzaud, S., Le Tilly, V., 2018. Threat of plastic ageing in marine environment. Adsorption/desorption of micropollutants. Mar. Pollut. Bull. 127, 684–694. https://doi.org/10.1016/J.MARPOLBUL.2017.12.059.
Kedzierski, M., Le Tilly, V., Bourseau, P., Bellegou, H., César, G., Sire, O., Bruzaud, S., 2016. Microplastics elutriation from sandy sediments: a granulometric approach. Mar. Pollut. Bull. 107, 315–323.
Kim, I.-S., Chae, D.-H., Kim, S.-K., Choi, S., Woo, S.-B., 2015. Factors influencing the spatial variation of microplastics on high-tidal coastal beaches in Korea. Arch. Environ. Contam. Toxicol. 69, 299–309. https://doi.org/10.1007/s00244-015-0155-6.
Kukulka, T., Proskurowski, G., Morét-Ferguson, S., Meyer, D.W., Law, K.L., 2012. The effect of wind mixing on the vertical distribution of buoyant plastic debris. Geophys. Res. Lett. 39https://doi.org/10.1029/2012GL051116. n/a-n/a.
Laglbauer, B.J.L., Franco-Santos, R.M., Andreu-Cazenave, M., Brunelli, L., Papadatou, M., Palatinus, A., Grego, M., Deprez, T., 2014. Macrodebris and microplastics from beaches in Slovenia. Mar. Pollut. Bull. 89, 356–366. https://doi.org/10.1016/j.marpolbul.2014.09.036.
Lavers, J.L., Bond, A.L., 2017. Exceptional and rapid accumulation of anthropogenic debris on one of the world's most remote and pristine islands. Proc. Natl. Acad. Sci. U. S. A. 114, 6052–6055. https://doi.org/10.1073/pnas.1619818114.
Liu, H., Tang, L., Liu, Y., Zeng, G., Lu, Y., Wang, J., Yu, J., Yu, M., 2019. Wetland-a hub for microplastic transmission in the global ecosystem. Resour. Conserv. Recycl. 142, 153–154. https://doi.org/10.1016/j.resconrec.2018.11.028.
Lots, F.A.E., Behrens, P., Vijver, M.G., Horton, A.A., Bosker, T., 2017. A large-scale investigation of microplastic contamination: abundance and characteristics of microplastics in European beach sediment. Mar. Pollut. Bull. 123, 219–226. https://doi.org/10.1016/j.marpolbul.2017.08.057.
Lozoya, J.P., Teixeira de Mello, F., Carrizo, D., Weinstein, F., Olivera, Y., Cedrés, F., Pereira, M., Fossati, M., 2016. Plastics and microplastics on recreational beaches in Punta del Este (Uruguay): unseen critical residents? Environ. Pollut. 218, 931–941. https://doi.org/10.1016/j.envpol.2016.08.041.
Martellini, T., Guerranti, C., Scopetani, C., Ugolini, A., Chelazzi, D., Cincinelli, A., 2018. A snapshot of microplastics in the coastal areas of the Mediterranean Sea. TrAC - Trends Anal. Chem. 109, 173–179. https://doi.org/10.1016/j.trac.2018.09.028.
Nel, H.A., Froneman, P.W., 2015. A quantitative analysis of microplastic pollution along the south-eastern coastline of South Africa. Mar. Pollut. Bull. 101, 274–279. https://doi.org/10.1016/J.MARPOLBUL.2015.09.043.
OECD, 2017. Tourism Policy Review of Mexico - Executive Summary.
Ogonowski, M., Gerdes, Z., Gorokhova, E., 2018. What we know and what we think we know about microplastic effects – a critical perspective. Curr. Opin. Environ. Sci. Health 1, 41–46. https://doi.org/10.1016/j.coesh.2017.09.001.
Ory, N.C., Sobral, P., Ferreira, J.L., Thiel, M., 2017. Amberstripe scad Decapterus muroadsi (Carangidae) fish ingest blue microplastics resembling their copepod prey along the coast of Rapa Nui (Easter Island) in the South Pacific subtropical gyre. Sci. Total Environ. 586, 430–437. https://doi.org/10.1016/j.scitotenv.2017.01.175.
Piñon-Colin, Tde J., Rodriguez-Jimenez, R., Pastrana-Corral, M.A., Rogel-Hernandez, E., Wakida, F.T., 2018. Microplastics on sandy beaches of the Baja California peninsula. Mexico. Mar. Pollut. Bull. 131, 63–71. https://doi.org/10.1016/J.MARPOLBUL.2018.03.055.
Purca, S., Henostroza, A., 2017. Presencia de microplásticos en cuatro playas arenosas de Perú. Rev. Peru. Biol. 24, 101. https://doi.org/10.15381/rpb.v24i1.12724.
Retama, I., Jonathan, M.P., Shruti, V.C., Velumani, S., Sarkar, S.K., Roy, P.D., Rodríguez-Espinosa, P.F., 2016. Microplastics in tourist beaches of Huatulco Bay, Pacific coast of southern Mexico. Mar. Pollut. Bull. 113, 530–535. https://doi.org/10.1016/j.marpolbul.2016.08.053.
Ryberg, M.W., Hauschild, M.Z., Wang, F., Averous-Monnery, S., Laurent, A., 2019. Global environmental losses of plastics across their value chains. Resour. Conserv. Recycl. 151, 104459. https://doi.org/10.1016/j.resconrec.2019.104459.
SECTUR, 2018. Vision Global del Turismo a Mexico.
SEMARNAT, Sde M.Ay R.N., 2019. Programa integral de playas. limpias [WWW Document]. Programa Integr. Play. limpias. URL https://apps1.semarnat.gob.mx:8443/dgeia/gob-mx/playas/pdf/acciones.pdf (consultado 8.25.19). .
de energía Sener, S., 2006. NMX-AA-120-SCFI-2006. Que establece los requisitos y especificaciones de sustentabilidad de calidad de playas. México.
Shim, W.J., Hong, S.H., Eo, S., 2018. Marine Microplastics: Abundance, Distribution, and Composition, Microplastic Contamination in Aquatic Environments. Elsevier Inc.https://doi.org/10.1016/b978-0-12-813747-5.00001-1.
Shruti, V.C., Jonathan, M.P., Rodriguez-Espinosa, P.F., Rodríguez-González, F., 2019. Microplastics in freshwater sediments of Atoyac River basin, Puebla City, Mexico. Sci. Total Environ. 654, 154–163. https://doi.org/10.1016/j.scitotenv.2018.11.054.
Siegfried, M., Koelmans, A.A., Besseling, E., Kroeze, C., 2017. Export of microplastics from land to sea. A modelling approach. Water Res. 127, 249–257. https://doi.org/10.1016/j.watres.2017.10.011.
Tiwari, M., Rathod, T.D., Ajmal, P.Y., Bhangare, R.C., Sahu, S.K., 2019. Distribution and characterization of microplastics in beach sand from three different Indian coastal

environments. Mar. Pollut. Bull. 140, 262–273. https://doi.org/10.1016/J.MARPOLBUL.2019.01.055.
Tsiota, P., Karkanorachaki, K., Syranidou, E., Franchini, M., Kalogerakis, N., 2018. Microbial Degradation of HDPE Secondary Microplastics: Preliminary Results. pp. 181–188. https://doi.org/10.1007/978-3-319-71279-6_24.
UNEP, 2006. Marine plastic debris & microplastics. America (NY) 23, 1–2. https://doi.org/10.2173/bna.44.
Vedolin, M.C., Teophilo, C.Y.S., Turra, A., Figueira, R.C.L., 2019. Spatial variability in the concentrations of metals in beached microplastics. s/f.. Mar. Pollut. Bull. 129, 487–493. https://doi.org/10.1016/j.marpolbul.2017.10.019.
Veerasingam, S., Mugilarasan, M., Venkatachalapathy, R., Vethamony, P., 2016a. Influence of 2015 flood on the distribution and occurrence of microplastic pellets along the Chennai coast. India. Mar. Pollut. Bull. 109, 196–204. https://doi.org/10.1016/j.marpolbul.2016.05.082.
Veerasingam, S., Saha, M., Suneel, V., Vethamony, P., Rodrigues, A.C., Bhattacharyya, S., Naik, B.G., 2016b. Characteristics, seasonal distribution and surface degradation features of microplastic pellets along the Goa coast, India. Chemosphere 159, 496–505. https://doi.org/10.1016/J.CHEMOSPHERE.2016.06.056.
Walker, T.R., Xanthos, D., 2018. A call for Canada to move toward zero plastic waste by reducing and recycling single-use plastics. Resour. Conserv. Recycl. 133, 99–100. https://doi.org/10.1016/j.resconrec.2018.02.014.
Wang, C., Zhao, L., Lim, M.K., Chen, W.Q., Sutherland, J.W., 2020. Structure of the global plastic waste trade network and the impact of China's import Ban. Resour. Conserv. Recycl. 153. https://doi.org/10.1016/j.resconrec.2019.104591.
Wen, X., Du, C., Xu, P., Zeng, G., Huang, D., Yin, L., Yin, Q., Hu, L., Wan, J., Zhang, J., Tan, S., Deng, R., 2018. Microplastic pollution in surface sediments of urban water areas in Changsha, China: abundance, composition, surface textures. Mar. Pollut. Bull. 136, 414–423. https://doi.org/10.1016/j.marpolbul.2018.09.043.
Wessel, C.C., Lockridge, G.R., Battiste, D., Cebrian, J., 2016. Abundance and characteristics of microplastics in beach sediments: insights into microplastic accumulation in northern Gulf of Mexico estuaries. Mar. Pollut. Bull. 109, 178–183. https://doi.org/10.1016/j.marpolbul.2016.06.002.
Wilkinson, J., Hooda, P.S., Barker, J., Barton, S., Swinden, J., 2017. Occurrence, fate and transformation of emerging contaminants in water: an overarching review of the field. Environ. Pollut. 231, 954–970. https://doi.org/10.1016/j.envpol.2017.08.032.
